# ICS/formoterol in the management of asthma in the clinical practice of pulmonologists: an international survey on GINA strategy

**DOI:** 10.1186/s40733-021-00067-z

**Published:** 2021-01-29

**Authors:** Álvaro A. Cruz, Sara Barile, Elena Nudo, Laura Brogelli, Patricia Guller, Alberto Papi

**Affiliations:** 1 Fundação ProAR and Federal University of Bahia, Salvador, Brazil; 2 Chiesi Farmaceutici SpA, Parma, Italy; 3 Polistudium srl, Milan, Italy; 4 University of Ferrara, Ferrara, Italy

**Keywords:** Inhaled corticosteroid, Short-acting beta-2 agonist, Long-acting beta-2 agonist, Asthma

## Abstract

**Background:**

The treatment with short-acting beta-2 agonists (SABA) alone is no longer recommended due to safety issues. Instead, the current Global Initiative for Asthma (GINA) Report recommends the use of the combination of inhaled corticosteroids (ICS) with the rapid/long-acting beta-2 agonist formoterol, although the use in steps 1 and 2 is still off-label in the EU and in many countries. It is important to understand clinicians’ knowledge and opinions on the issue with the ultimate goal to encourage the implementation of the new approach in clinical practice.

**Methods:**

We performed an international survey, directed to pulmonologists interested in the management of patients with asthma.

**Results:**

Most participants reported that SABA alone should not be used in GINA Step 1 asthma treatment. As-needed low-dose ICS/formoterol combination to patients in step 1, and as-needed low-dose ICS/formoterol as reliever therapy in any step were found to be of current use prescribed in their real-life settings. SABA alone was still prescribed to a proportion of patients, although the pulmonologists’ opinion was that it should no longer be used.

**Conclusions:**

Most specialists are up to date and understand the relevance of the changes in GINA reports from 2019. Nevertheless, dissemination and implementation of GINA novel management strategy is still needed.

**Supplementary Information:**

The online version contains supplementary material available at 10.1186/s40733-021-00067-z.

## Background

The Global Initiative for Asthma (GINA) critically revises evidence on asthma management yearly and provides a structured set of recommendations [1]. The 2020 GINA Report maintains a major change in the management recommendations, which were introduced in 2019, in relation to pharmacological treatment [2]. The treatment with short-acting beta-2 agonists (SABA) alone, which has historically been the therapy of choice for mild patients (step 1), and common reliever treatment for other stages are no longer recommended due to safety issues, since the risk of exacerbations has been reported to be increased by their regular or frequent use [1]. Usage of SABAs in the absence of effective anti-inflammatory treatment, was associated with increased risk of asthma exacerbations, hospitalization and mortality due to asthma [2–5].

Therefore, current GINA Report recommends use of the combination of inhaled corticosteroids (ICS) with the rapid/long-acting beta-1 agonist formoterol instead of SABA alone, including in steps 1 and 2 of treatment, where the use is currently still off-label in the EU and many countries.

In line with the same concept of including an ICS in the rescue medication, another major change that was introduced in 2019 is that reliever therapy with as-needed low-dose ICS/formoterol is now recommended as the preferred rescue option for any asthma step, except when patients are using another ICS–long-acting beta-2 agonist combination as a controller [2].

Evidence for these recommendations was produced by large randomized controlled trials and by real-world studies. Two randomized, double-blind, placebo-controlled trials showed the efficacy and safety of budesonide–formoterol as reliever therapy in the absence of regular maintenance treatment in patients with mild asthma [6, 7]. The three-way SYGMA 1 trial included patients on GINA step 2 treatment and indicated that as-needed budesonide–formoterol combination was superior to as-needed SABA and provided a non-inferior effect on annual rate of exacerbation reduction, with a lower exposure to ICSs, when compared to a maintenance ICS regimen [6]. The results of SYGMA 2 trial, including the same patient population, also indicated non-inferiority of the as-needed budesonide–formoterol combination compared to the maintenance ICS plus as-needed SABA regimen in reducing the exacerbation rate in patients with mild asthma [7].

More recently, open-label randomized controlled trials confirmed the findings of the SYGMA studies. The Novel START (Novel Symbicort Turbuhaler Asthma Reliever Therapy) trial, investigated budesonide–formoterol reliever therapy used on an as-needed basis in adults with mild asthma who had been treated with only as-needed SABA, compared with ICS maintenance therapy plus as-needed SABA or as-needed SABA only. This was an open-label study in the clinical practice setting and demonstrated the external validity of SYGMA 1 and 2 [8]. The open-label, randomized PRACTICAL study, carried out in 15 primary care or hospital-based clinical units and primary care practices, showed that incidence of severe exacerbations was lower with as-needed budesonide–formoterol than with maintenance budesonide plus terbutaline as needed [9].

As the recent GINA Reports introduced landmark changes in recommendations for asthma management, it is important to understand clinicians’ knowledge and opinions on the issue with the ultimate goal to encourage the implementation of the new approach in clinical practice. In particular, these issues include the knowledge of current evidence on mild asthma treatment, the implementation of guidelines in clinical practice and the related difference across different clinical settings or in different geographic areas, with a particular emphasis on the acceptance of the changes in the 2019 GINA Report by clinicians.

As surveys are widely used to investigate physicians’ perspective in the management of asthma [10–13], we have performed an international survey, directed to pulmonologists interested in the management of patients with asthma, with the aim to investigate their opinion/behavior on the changes introduced in the GINA Report from 2019 and to assess the existence of any difference across countries.

### Participants and methods

#### Participant selection

Pulmonologists from different countries, identified in a proprietary database, were invited to answer the survey. Different countries were involved to gather a sample suitable to address the research question with a global approach. Clinicians could be invited if they had more than 5 years of clinical experience in respiratory disease, patients with asthma represented at least 25% of their practice in the last month, and at least 80% of their patients were adult (≥18 years) subjects.

## Methods

The survey was developed with the assistance of an independent third party, with broad experience in market research in the pharmaceutical setting (DoxaPharma, Milan, Italy), and then shared it with the authors for discussions via several online meetings until a final agreement was reached. The questionnaire was then delivered online via a computer-assisted web interview. The questionnaire contained 42 questions. Open and closed (multiple choice, with either single or multiple permitted answers) questions were included. Interviews were anonymous. The level of agreement was measured by 5-point Likert scale. Data were analyzed by descriptive statistics.

The English version of the survey questionnaire is presented as supplementary material.

## Results

### Sample description

The survey was conducted online from January to February 2020. A total of 160 pulmonologists based in Italy, Germany, The Netherlands, Brazil, China and Russia answered the questionnaire. Ten pulmonologists were from The Netherlands, 30 from each one of the other countries. In total, 59% of participants were older than 46 years, and 53% had more than 15 years of clinical experience as pulmonologists (Table 1).

**Table 1:** Participants

Participants feature	
Specialty	Pulmonologists (*n* = 160)
Countries	The Netherlands, *n* = 10 Italy (*n* = 30) Germany (*n* = 30) Brazil (*n* = 30) China (*n* = 30) Russia (*n* = 30)
5–15 years of clinical experience in respiratory diseases	48%
More than 15 years of clinical experience in respiratory diseases	53%
Asthma in respondents’ patients was:	
• Mild	27% of patients
• Moderate	43% of patients
• Severe	30% of patients

On average, asthma patients represented 47% of patient’s visits each month. Among patients with asthma, 27% had mild disease, 43% moderate disease, and 30% severe asthma, as expected in a specialist care setting. Follow-up of patients with asthma was performed on average every 3–4 months by 45% of participants (with country variations from 43% in Italy to 57% in Germany), based on severity.

### Current practice

The most likely prescribed therapy for patients in GINA step 1 was as-needed low-dose ICS/formoterol for 44% (up to 57% in Brazil) of clinicians. As-needed low-dose ICS/formoterol was reported to be prescribed to an average of 37% of patients (up to 49% in Brazil), followed by as-needed low-dose ICS. Among patients in step 1, who were prescribed low-dose ICS/formoterol, 51% received a prescription of budesonide–formoterol, and 44% received a prescription of beclometasone dipropionate–formoterol. In total, 95% of physicians followed this strategy proposed by GINA in step 1, and checked patients’ adherence to the prescribed therapy, either by an interview at follow-up visits or by asking patients to fill in a diary. Diaries are most often used in China (67%) and never used in The Netherlands.

**Fig. 1:**
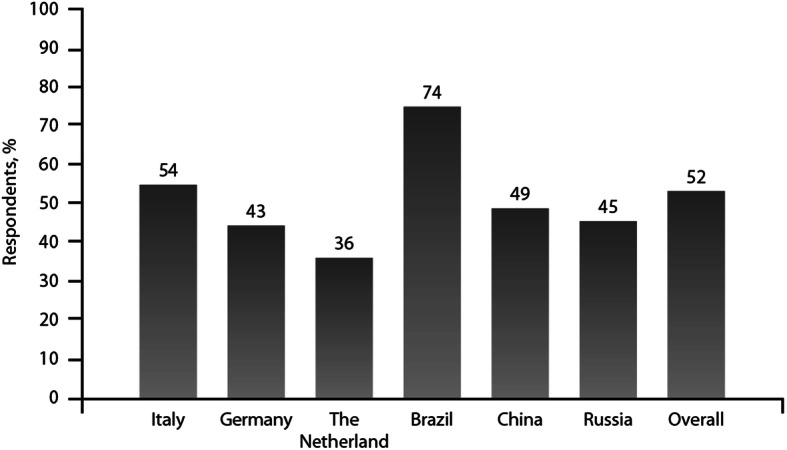
Low-dose ICS formoterol as the most frequent prescription of a reliever: national variation

The reliever therapy most likely to be prescribed in any step was as-needed low-dose ICS/formoterol for 61% of clinicians. It was currently prescribed to 52% of patients (with variations from 36% in The Netherlands to 74% in Brazil (Fig. 1). Among patients prescribed as-needed low-dose ICS/formoterol as a reliever, 50% received budesonide–formoterol and 48% beclometasone dipropionate–formoterol, whereas the other 2% used other formulations.

As-needed SABA was prescribed to 47% of patients, and 38% of physicians considered it as the reliever therapy they would prescribe most often.

A total of 82% of pulmonologists reported that identification of asthma phenotypes from the first stage of diagnosis was important to guide tailored therapeutic approaches.

Assessment of risk factors for exacerbations was reported to be performed by 33% of clinicians only when an exacerbation occurs.

### Drivers to treatment decision

Some questions investigated criteria used by participants to prescribe a therapeutic regimen for asthma, in their clinical practice. Treatment decisions in asthma patients were based mainly on clinical outcomes, such as symptom control, which was the most important driver in 48% of cases. Characteristics of the drug, such as safety and cost, were also important criteria; on the contrary, patient’s characteristics, such as the physical fitness, the level of adherence, comorbidities, history, and economic situation, were rarely considered (Table 2).

**Table 2:** Main criteria used by participants to prescribe a therapeutic regimen for asthma

Criterium	Pulmonologists (%)
Symptom control	48
Asthma clinical situation	33
Features of the drug	10
Characteristics of the patient	7
Route of administration of the medication	1
Other	2

### Attitude toward guidelines

**Fig. 2:**
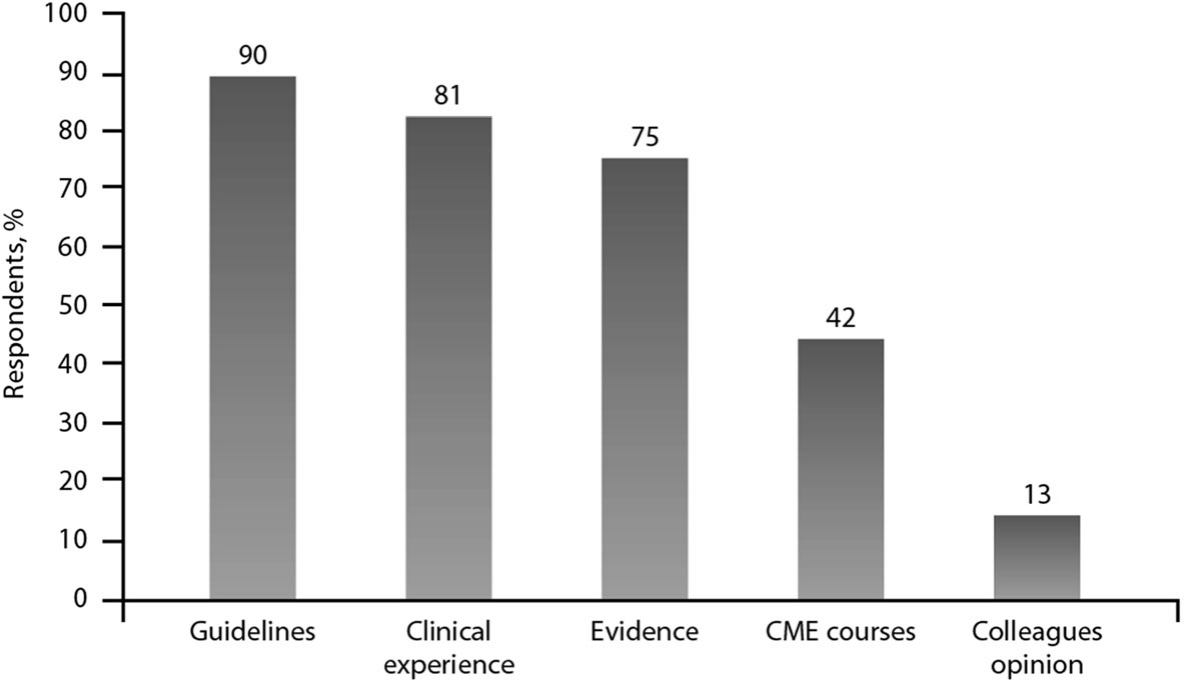
Points of reference in prescription choice

Guidelines were the main source of reference in prescription choice (90% of pneumologists), followed by past clinical experience (81%) and published evidence (75%) (Fig. 2). Physicians who did not rely on guidelines trusted past clinical experience in 42% of cases. Past clinical experience was more relevant for Brazilian clinicians than for those from other countries. It was a primary driver of choice of treatment for 47% of participants from Brazil, 27% from Italy, 23% from Germany, 13% from China, 10% from The Netherlands and Russia.

**Fig. 3:**
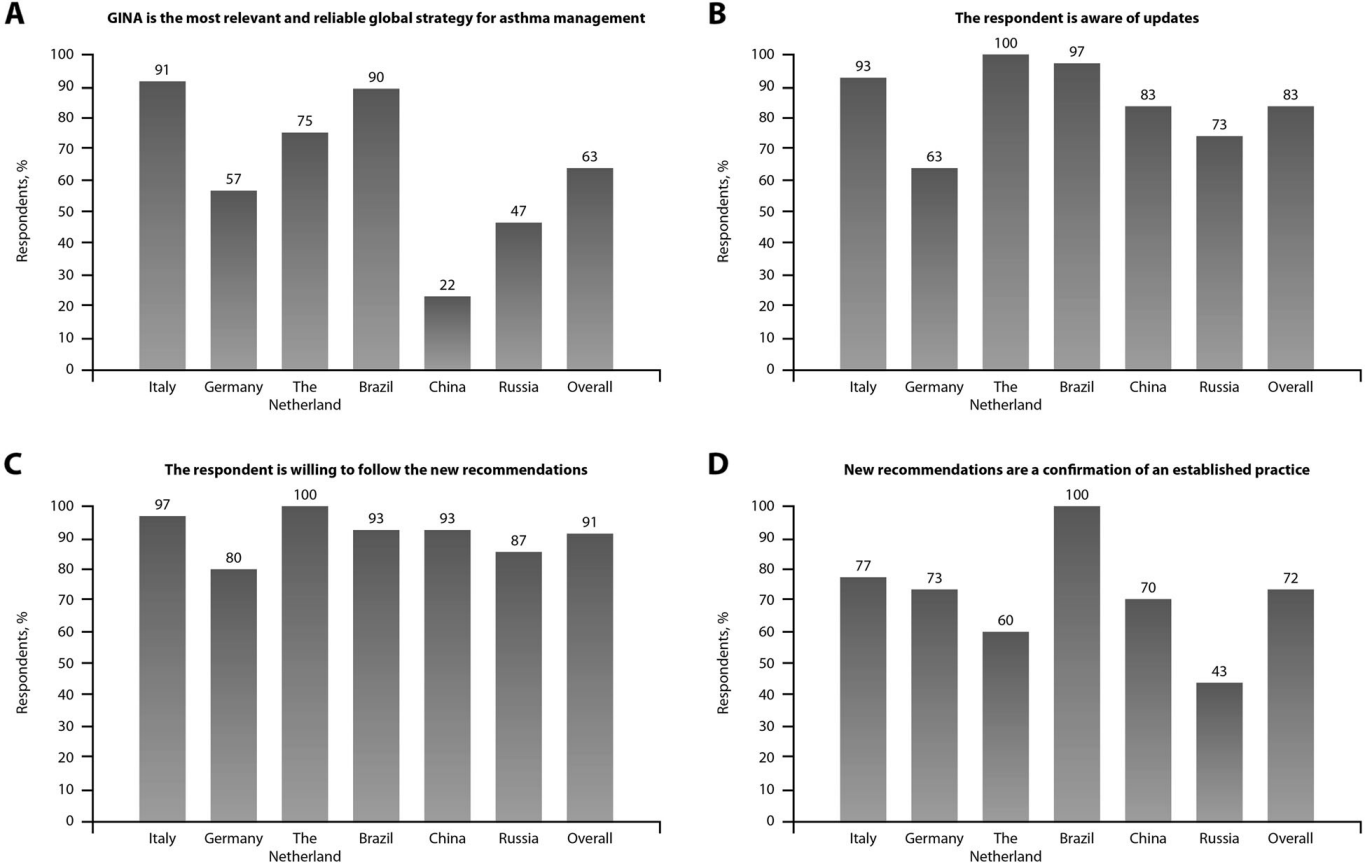
Attitude towards GINA Report. **a**: Reliability of GINA Report versus guidelines; **b**: Awareness of updates to GINA Report, **c**: Willingness to adhere to the new GINA strategy, **d**: Agreement between new GINA strategy and current clinical practice

GINA Reports were considered the most relevant and reliable management strategy (mainly for Italian and Brazilian pulmonologists, with 91 and 90% of answers, respectively). Overall, 83% of participants were aware of updates in the 2019 GINA Report. The use of ICS/formoterol in mild asthma was considered as the main change introduced in 2019 by 34% of participants, while the recommendation not to use SABA in monotherapy was the main key change for 30% of clinicians. Overall, 91% of interviewed pulmonologists were willing to follow the new recommendations (Fig. 3). For 72% of participants (with a wide variability according to countries, from 100% in Brazil to 43% in Russia) the new recommendations represented a confirmation of an established practice, while for only 22% of physicians they required a radical change of the clinical approach to asthma.

Specifically, prescribing as-needed low-dose ICS/formoterol to the patients in GINA step 1 was an established practice for 76/138 (55%) of pulmonologists (from 14% [1/7] in The Netherlands to 20/30 [67%] in Brazil), and a recent change for the remaining 45% physicians. In the latter group of respondents, the recent change of clinical behavior was acknowledged to have been adopted to adhere to GINA Report in 74% of cases and based on results from published clinical studies in 21% of cases.

Prescribing as-needed low-dose ICS/formoterol as reliever therapy in any step was an established practice for 59% of subjects (from 33% in The Netherlands to 80% in Brazil).

When asked about their patients’ attitude toward asthma treatment, 60% of physicians (90% in The Netherlands and in Brazil, 67% in China, 60% in Germany, 40% in Italy and 33% in Russia) thought that patients in GINA steps 1 and 2 may adhere better to ICS/formoterol treatment with as-needed therapy than with a regular therapy. On the contrary, 40% (67% in Russia, 60% in Italy, 40% in Germany, 33% in China, 10% in The Netherlands and Brazil) of respondents reported that this therapy would be better adhered if this treatment would be prescribed as maintenance.

## Discussion

We conducted an international survey among pulmonologists to investigate the attitude towards the key changes in asthma therapy introduced in the 2019 GINA Report based on evidence. These specialists are, globally, the main reference for therapeutic decisions, with a pivotal role. GPs and other healthcare professionals are, indeed, involved in asthma management, with different roles according to the country’s health system; they were excluded to obtain a homogeneous sample.

In answering our survey, most participants reported that SABA alone in GINA step 1 should not be used. This result was in agreement with published evidence, which most clinicians seemed to be well acquainted with [14]. In addition, prescribing as-needed low-dose ICS/formoterol combination to patients in GINA step 1, and as-needed low-dose ICS/formoterol as reliever therapy in any step were found to be current use in the real-life setting. The use of ICS/formoterol in GINA step 1 is commonly practiced in Italy and Brazil, less often in the other countries (Brazil 57%, Italy 47%, China 43%, Germany and The Netherlands 40%, Russia 37% of participants); the risk of low patient’s adherence seems not to be a barrier to the use of ICS/formoterol on demand. The approach to mild asthma management here described was in agreement with available evidence and clinicians seemed to be updated and to understand the relevance of new GINA recommendations [6, 7, 15–18].

Many pulmonologists acknowledged that GINA Reports were their point of reference for clinical decision, but the proportion of physicians in this group varied in the countries within the survey. Nonetheless, even when GINA Reports were not the main reference, most pulmonologists had adopted the recommended key changes in therapy choice, based either on published literature or on clinical experience. Indeed, this latter item can be considered as a composite of scientific evidence, personal experience and colleagues’ experience.

Among guidelines, the GINA Report is the most used one for pulmonologists, but many physicians also rely on local guidelines. In addition, pulmonologists answered that attention to the GINA Report would increase in the future; such an attitude may suggest that clinicians are evaluating the recommendations, studying the evidence and implementing the change, accordingly; moreover, participation in the survey, focusing on relevant issues may itself have an educational effectiveness. Despite the pulmonologists’ opinion that it should no longer be used, SABA alone is still broadly used, suggesting that dissemination and implementation of GINA Strategy changes are much needed.

A previous cross-sectional study in the general practice, in Italy, had shown that although GINA Reports were considered relevant for treatment decision, adherence to treatment was low for patients with mild asthma and was higher for patients with moderate or severe asthma [19]. These observations agree with the findings in our survey that patients in GINA step 1 were not usually willing to adhere to continuous controller therapy, suggesting that patients with mild disease often underestimate their illness potential to harm them.

Phenotype identification was considered an early step in patient’s evaluation, and necessary for treatment decision in severe asthma, so that therapies can be better targeted toward disease-specific features [20–24]. It is currently believed that successful therapy of asthma requires better definition of underlying pathogenesis, to tailor individualized, evidence-based and more precise therapy options [21].

Finally, answers to the survey suggested that adherence to guidelines can be improved in some countries, such as The Netherlands and Germany. In some instances, it might be important to spread in-depth information about the pharmacological profile of different ICSs. In addition, it is necessary to further understand the barriers of implementation of changes, which were found there. Although a survey is a useful method for collecting data on the needed changes, it has a limitation in the potential for understanding the barriers to implementation of changes, and an additional in-depth qualitative semi-structured interview would be needed.

A limitation of our survey may be linked to different organizations in the health system of the countries involved; a homogeneous sample of pulmonologists was interviewed, omitting family physicians and nurses who may have relevant roles in certain countries but not in others.

Statistically relevant comparisons among countries were not the objective of the study. A global sample of pulmonologists was investigated and differences among countries could only be reported as descriptive data on the sample.

In conclusion, the survey showed that the changes in GINA Strategy for asthma management from 2019 validated an established practice in some countries, while for a minority of physicians they required a radical change of the clinical approach to asthma. Most pulmonologists take into account or are willing to take into account in their practice the fundamental changes proposed by GINA.^1^ The greatest change in the approach to asthma management introduced by the 2019 GINA Report is supported by relevant evidence, and pulmonologists appear to acknowledge the scientific background of the report and adopt it.

## Supplementary Information

**Additional file 1.** Questionnaire.

## Data Availability

The datasets used and/or analysed during the current study are available from the corresponding author on reasonable request.

## Abbreviations

GINA: Global Initiative for Asthma
ICS: Inhaled corticosteroid
SABA: Short-acting beta-2 agonists
