# Supplementary material for: ICS/formoterol in the management of asthma in the clinical practice of pulmonologists: an international survey on GINA strategy
Source: Asthma Res Pract. 2021 Jan 29;7:1. doi: 10.1186/s40733-021-00067-z (PMC7844897; doi:10.1186/s40733-021-00067-z)
Supplement: Supplementary file 1 — Additional file 1. Questionnaire. [file 40733_2021_67_MOESM1_ESM.docx]

**QUESTIONNAIRE**

**S0. Country**

1. ITALY
2. GERMANY
3. THE NETHERLANDS
4. RUSSIA
5. CHINA
6. BRAZIL

**S1. What is your primary specialty?**

1. Pneumology

2. Other 🡪 ***THANK AND CLOSE***

**S2. How long have you been focused on your primary specialty?**

1. Less than 5 years 🡪 ***THANK AND CLOSE***
2. 5–15 years
3. More than 15 years

**S3. Do you personally treat and manage patients with ASTHMA?**

1.Yes

2. No 🡪 ***THANK AND CLOSE***

**S4. Do you personally prescribe treatments for ASTHMA?**

1. Yes

2. No 🡪 ***THANK AND CLOSE***

**S5. How many patients do you see in an average month for any kind of respiratory pathology?**

________________ no. of patients/month

**S6. And how many of these ____(S5) patients are affected with ASTHMA?**

________________ no. of ASTHMATIC patients/months

***IF LESS THAN 25% PATIENTS 🡪 THANK AND CLOSE***

**S7. Of these _____ (*S6*) patients, what % is affected with...**

1. /___________ / % MILD ASTHMA
2. /___________ / % MODERATE ASTHMA
3. /___________ / % SEVERE ASTHMA

Total 100%

**S8. Considering the age of the asthma patients you see in an average month, approximately what percentage of asthma patients are…?**

1. /___________ / % Less than 5 years old
2. /___________ / % 6–11 years old
3. /___________ / % 12–17 years old
4. /___________ / % 18 years and older

*Total 100%*

***IF code 4 < 80% 🡪 THANK AND CLOSE***

***Doctor, we would like you to focus your attention on the treatment of your asthma patients.***

**Q1. Which are your top 3 drivers of choice when it comes to prescribing a drug to patients affected with ASTHMA?**

1_______________________________________

2_______________________________________

3_______________________________________

**Q2. Which of the following clinical practice-related factors most determine which treatment for asthma you prescribe for your patients? Rank the following factors in order of importance**

*ROTATE ITEM 1-5*

1. Past clinical experiences
2. National/International guidelines
3. Colleague’s recommendations
4. Available published evidence
5. Continuing Medical Education (CME) courses
6. Other clinical practice-related factors 🡪 please specify___________________

*IF Q2 CODE 2 in 1° or 2° position*

**Q3. Which guidelines do you consider most relevant in your treatment choice for asthma?**

*IF Q2 NOT CODE 2 in 1° or 2° position*

**Q4. Why don’t you consider the guidelines in your treatment choice as most relevant?**

**Q5. Considering the patients with asthma you see in an average month, what percentage are in the different steps of treatments recommended by the GINA guidelines?**

1. /___________ / % STEP 1 (symptoms <2/month and no exacerbation risk factors)
2. /___________ / % STEP 2 (symptoms 0-1 days/week)
3. /___________ / % STEP 3 (asthma is uncontrolled on low dose ICS)
4. /___________ / % STEP 4 (asthma is uncontrolled on low dose ICS-LABA)
5. /___________ / % STEP 5 (uncontrolled symptoms and/or exacerbations despite step 4 treatment)

Total 100%

**Q6. Considering the patients with asthma you see in an average month that are in STEP 4 of GINA guidelines (asthma is uncontrolled on low dose ICS-LABA), what % of your patients are taking the following CONTROLLER THERAPIES (e.g. to be taken regularly every day):**

*ROTATE ITEM 1-4*

1. /___________ / % Medium-dose ICS-LABA
2. /___________ / % High-dose ICS-LABA
3. /___________ / % High-dose ICS-LABA + tiotropium
4. /___________ / % High-dose ICS-LABA + LTRA
5. /___________ / % Other (please specify) ____________________________________

**Q7. Considering the patients with asthma you see in an average month that are in STEP 5 of GINA guidelines, what % of your patients are taking the following CONTROLLER THERAPIES (e.g. to be taken regularly every day):**

*ROTATE ITEM 1-6*

1. /___________ / % High-dose ICS-LABA
2. /___________ / % High-dose ICS-LABA + tiotropium
3. /___________ / % High-dose ICS-LABA + anti igE
4. /___________ / % High-dose ICS-LABA + anti IL5/5R
5. /___________ / % High-dose ICS-LABA + anti IL4R
6. /___________ / % High-dose ICS-LABA + OCS
7. /___________ / % Other (please specify) ____________________________________

**Q11. Please indicate which of the following is the CONTROLLER THERAPY that you are most likely to prescribe for patients that are in STEP 1 of GINA guidelines (i.e. patients with symptoms <twice/month and no risk factors for exacerbations):**

1. Low-dose ICS as needed
2. Low-dose ICS/formoterol as needed
3. Low-dose ICS taken whenever SABA is taken
4. No controller therapy for patients in STEP 1
5. Other, please specify____________________________________

**Q12. Considering the patients with asthma you see in an average month that are in STEP 1 of GINA guidelines, what % of your patients are taking the following CONTROLLER THERAPIES:**

1. /___________ / % Low-dose ICS as needed
2. /___________ / % Low-dose ICS/formoterol as needed
3. /___________ / % Low-dose ICS taken whenever SABA is taken
4. /___________ / % No controller therapy
5. /___________ / % Other (please specify) ____________________________________

*IF Q12 CODE 2 > 0*

**Q13. You said to prescribe “*Low-dose ICS/formoterol as needed”* as CONTROLLER THERAPY to your_____% (Q12.2) patients in STEP 1 of GINA guidelines, to what % of these patients is prescribed the following drug as CONTROLLER THERAPY?**

1. BDP/FF (Beclomethasone dipropionate/Formoterol) /___________ / %
2. BUD/FF (Budesonide/Formoterol) /___________ / %
3. Another ICS-Formoterol (please specify) __________ /___________ / %

Total 100%

*IF Q12 CODE 2 > 0*

**Q14. You said to prescribe “*Low-dose ICS/formoterol as needed”* to the patients in STEP 1 of GINA guidelines, this therapeutical attitude is:**

1. An established practice

2. A recent change

*IF Q14 CODE 2*

**Q15. What is the reason why for this change?**

1. New guidelines
2. Colleague’s recommendations
3. Continuing Medical Education (CME) courses
4. New published evidence
5. Last update of GINA guidelines (in 2019)
6. Other factors (please specify) ___________________________

**Q16. Please indicate which of the following is the RELIEVER THERAPY that you are most likely to prescribe for asthmatic patients independently from the step of treatment:**

1. SABA as needed
2. Low-dose ICS/formoterol as needed
3. Other (please specify) ____________________________________

**Q17. Considering the patients with asthma you see in an average month, what % of your patients are taking the following RELIEVER THERAPIES:**

1. /___________ / % SABA as needed
2. /___________ / % Low-dose ICS/formoterol as needed
3. /___________ / % Other (please specify) ________________________________

*IF Q17 CODE 2 > 0*

**Q18. You said to prescribe “*Low-dose ICS-formoterol as needed”* as RELIEVER THERAPY to your ___(Q17.2) patients, what % of these patients is prescribed the following treatments as RELIEVER THERAPY?**

1. BDP/FF (Beclomethasone dipropionate/Formoterol) /___________ / %
2. BUD/FF (Budesonide/Formoterol) /___________ / %
3. Another ICS-Formoterol (please specify) __________ /___________ / %

Total 100%

*IF Q17 CODE 2 > 0*

**Q19. You said to prescribe “*Low-dose ICS-formoterol as needed”* as RELIEVER THERAPY, this therapeutical attitude is:**

1. An established practice

2. A recent change

*IF Q19= CODE 2*

**Q20. What is the reason why for this change?**

1. New guidelines
2. Colleague’s recommendations
3. Last update of GINA guidelines (in 2019)
4. New published evidence
5. Continuing Medical Education (CME) courses
6. Other factors (please specify) ___________________________

**Q21. Recently (in 2019), the GINA guidelines have been updated. Are you aware of this novelty?**

1. Yes
2. No

*IF Q21=YES code 1*

**Q22. Could you please list the 2 major changes in the 2019 GINA guidelines?**

___________________________________________________________________________________

*IF Q21=NO code 2 SHOW THIS PARAGRAPH*

**The new GINA guidelines recommend using as-needed low-dose ICS-formoterol in step 1 as the CONTROLLER treatment and to use as-needed low-dose ICS-formoterol as the PREFERRED RELIEVER THERAPY in every step of treatment**

*TO ALL*

**Q23. Would you follow the GINA 2019 guidelines recommendation, which indicate to use as-needed low-dose ICS-formoterol in step 1 as the PREFERRED CONTROLLER CHOICE to prevent exacerbations and control symptoms?**

1. Yes
2. No 🡪 **b.** **Why not**?___________________
3. I have no information yet about it

**Q24. Would you follow the GINA 2019 guidelines recommendation, which indicate to use as-needed low-dose ICS-formoterol as the PREFERRED RELIEVER THERAPY in every step of treatment?**

1. Yes
2. No 🡪 **b.** **Why not**?___________________
3. I have no information yet about it

**Q25. In relation to the use of as-needed low-dose ICS-formoterol as the PREFERRED RELIEVER THERAPY, please rank each of the below on the following patients’ characteristics. Please use a scale of 1–5 where: 1 = “Least preferred” and 5=“Most preferred”.**

|  | **Least preferred** | **Somewhat preferred** | **Neutral/no opinion** | **Preferred** | **Most preferred** |
| --- | --- | --- | --- | --- | --- |
| 1. **Non-adherent patients** |  |  |  |  |  |
| 1. **Severe asthma patients** |  |  |  |  |  |
| 1. **Obese patients** |  |  |  |  |  |
| 1. **Patients with respiratory comorbidities such as COPD or pulmonary disease** |  |  |  |  |  |
| 1. **Patient with respiratory depression** |  |  |  |  |  |
| 1. **Patients >75 years of age** |  |  |  |  |  |
| 1. **Mild asthma patients** |  |  |  |  |  |
| 1. **Adolescent asthma patients** |  |  |  |  |  |
| 1. **Sporting asthma patients** |  |  |  |  |  |

**Q26. Based on your clinical experiences, to what extent do you agree with the following statements? Please respond to each statement on a 1–5 scale where 1 = “strongly disagree” and 5 = “strongly agree”.**

|  | **Strongly disagree** | **Disagree** | **Neutral/**  **no opinion** | **Agree** | **Strongly agree** |
| --- | --- | --- | --- | --- | --- |
| 1. **As needed ICS-formoterol is the rescue option for patients treated with ICS-formoterol** |  |  |  |  |  |
| 1. **The use of low-dose ICS should be considered as early as step 1 and used through step 2 into step 3 as medium- to high-dose ICS** |  |  |  |  |  |
| 1. **In steps 4 and 5 of the treatment, the possible fixed triple therapy could be a better option rather than the add-on therapy with tiotropium** |  |  |  |  |  |
| 1. **In step 5 of the treatment, biologics are as step up from high dose ICS-LABA+ LAMA (add-on therapy)** |  |  |  |  |  |
| 1. **In step 5 of the treatment, biologics are alternative to high dose ICS-LABA + LAMA** |  |  |  |  |  |
| 1. **Identification of asthma phenotypes since the first stages of diagnosis is important to guide more tailored therapeutic approaches** |  |  |  |  |  |
| 1. **The use of as-needed low-dose ICS-formoterol as early as step 1 increases the use of anti-inflammatory CONTROLLER THERAPY** |  |  |  |  |  |

**Q27. Based on the new GINA 2019 guidelines recommendation, how will your prescriptions change as the PREFERRED CONTROLLER CHOICE for patients in step 1:**

|  | **BDP-FF**  **(**Beclomethasone dipropionate/Formoterol | **BUD-FF**  (Budesonide/Formoterol) | **Salbutamol** |
| --- | --- | --- | --- |
| 1. **Will significantly increase** | 1 | 1 | 1 |
| 1. **Will slightly increase** | 2 | 2 | 2 |
| 1. **Will remain the same** | 3 | 3 | 3 |
| 1. **Will slightly decrease** | 4 | 4 | 4 |
| 1. **Will significantly decrease** | 5 | 5 | 5 |

**Q30. On the basis of the new GINA 2019 guidelines recommendation, how will your prescriptions change as the RELIEVER THERAPY for patients in every step of the GINA recommendations:**

|  | **BDP-FF** | **BUD-FF** | **Salbutamol** |
| --- | --- | --- | --- |
| 1. **Will significantly increase** | 1 | 1 | 1 |
| 1. **Will slightly increase** | 2 | 2 | 2 |
| 1. **Will remain the same** | 3 | 3 | 3 |
| 1. **Will slightly decrease** | 4 | 4 | 4 |
| 1. **Will significantly decrease** | 5 | 5 | 5 |

**Q33. Thinking about the patients in step 1 GINA guidelines, in case of prescription of low-dose ICS-formoterol, how do you evaluate these drugs on a 1–5 scale where 1 = “the product doesn’t have the feature at all” and 5 = “the product has the feature strongly”.**

|  | **BDP-FF** | **BUD-FF** |
| --- | --- | --- |
| 1. **It has a good efficacy** |  |  |
| 1. **It has a good tolerability** |  |  |
| 1. **It is supported by valid scientific literature** |  |  |
| 1. **It ensures better patient adherence** |  |  |
| 1. **It has an easy-to-use device** |  |  |
| 1. **I have more experience with it** |  |  |

**Q34. On average, how often do you see patients affected with ASTHMA for follow-up purposes?**

Every ___________________months

**Q35. Risk factors are assessed in patients with controlled symptoms:**

1. At the same frequency as in patients with uncontrolled symptoms
2. Only in case of exacerbation

**Q36. When you are deciding to initiate a CONTROLLER THERAPY, asthma phenotyping is :**

1. Not necessary
2. Useful
3. Very important
4. To be considered in relation to disease severity

**Q37. When you start a CONTROLLER THERAPY in step 1, do you check adherence to treatment?**

1. Yes, with control visits every _____ month/months
2. Yes, asking the patient to have a diary
3. Yes, in other way, please specify ___________________
4. No

**Q38. Do you think that use of SABA as RELIEVER in steps 1–2 may is associated to low adherence to a controller therapy?**

1. Yes
2. No

**Q39. Do you think that in steps 1 and 2 adherence to ICS-formoterol may be higher with…**

1. As needed therapy
2. Maintenance therapy

**Q40. To what extent do you think that the new GINA 2019 guidelines have been applied among your colleagues in your country?**

1. Very much
2. Quite
3. Somewhat
4. Not at all

**Q41. What do you think that the new recommendations of GINA 2019 guidelines represent for your clinical practice?**

1. A radical change
2. A confirmation of an established practice
3. A change that I don’t agree with

**Q42. Considering your mild asthma patients, what % of patients do you think prefer the use of ICS in association with a rapid acting bronchodilator as needed (in the absence of regular treatment) compared to regular, daily maintenance treatment plus as needed SABA?**

| 1. /___________ / % | MILD asthma patients that prefer ICS in association with rapid acting bronchodilator as needed (without regular treatment) |
| --- | --- |
| 1. /___________ / % | MILD asthma patients that prefer regular/daily maintenance treatment + as needed SABA |
| Total 100% |  |

***Thank you for the collaboration***
